# Single-cell gene expression analysis reveals *β*-cell dysfunction and deficit mechanisms in type 2 diabetes

**DOI:** 10.1186/s12859-018-2519-1

**Published:** 2018-12-31

**Authors:** Lichun Ma, Jie Zheng

**Affiliations:** 1 Biomedical Informatics Lab, School of Computer Science and Engineering, Nanyang Technological University, Singapore, 639798 Singapore; 2 School of Information Science and Technology, ShanghaiTech University, Shanghai, 201210 China

**Keywords:** Single-cell, Hyperglycaemia, Type 2 diabetes, *β*-cell dysfunction, *β*-cell deficit, Insulin expression, Apoptosis, Oxidative stress

## Abstract

**Background:**

Type 2 diabetes (T2D) is one of the most common chronic diseases. Studies on T2D are mainly built upon bulk-cell data analysis, which measures the average gene expression levels for a population of cells and cannot capture the inter-cell heterogeneity. The single-cell RNA-sequencing technology can provide additional information about the molecular mechanisms of T2D at single-cell level.

**Results:**

In this work, we analyze three datasets of single-cell transcriptomes to reveal *β*-cell dysfunction and deficit mechanisms in T2D. Focused on the expression levels of key genes, we conduct discrimination of healthy and T2D *β*-cells using five machine learning classifiers, and extracted major influential factors by calculating correlation coefficients and mutual information. Our analysis shows that T2D *β*-cells are normal in insulin gene expression in the scenario of low cellular stress (especially oxidative stress), but appear dysfunctional under the circumstances of high cellular stress. Remarkably, oxidative stress plays an important role in affecting the expression of insulin gene. In addition, by analyzing the genes related to apoptosis, we found that the TNFR1-, BAX-, CAPN1- and CAPN2-dependent pathways may be crucial for *β*-cell apoptosis in T2D. Finally, personalized analysis indicates cell heterogeneity and individual-specific insulin gene expression.

**Conclusions:**

Oxidative stress is an important influential factor on insulin gene expression in T2D. Based on the uncovered mechanism of *β*-cell dysfunction and deficit, targeting key genes in the apoptosis pathway along with alleviating oxidative stress could be a potential treatment strategy for T2D.

**Electronic supplementary material:**

The online version of this article (10.1186/s12859-018-2519-1) contains supplementary material, which is available to authorized users.

## Background

Type 2 diabetes (T2D) is one of the major causes of death worldwide [1]. It is characterized by insulin resistance and impaired insulin secretion [2, 3]. Insulin resistance denotes declined insulin sensitivity in insulin targeted cells or tissues, while insufficient insulin secretion is related to pancreatic *β*-cell dysfunction and the loss of *β*-cell mass [4, 5]. *β*-cells are located in the islets of Langerhans, i.e. endocrine regions of pancreas. The main function of *β*-cells is to synthesize, store and secrete insulin, which is a peptide hormone and takes effects in decreasing the blood glucose level. It is reported that *β*-cell function declines even before the diagnosis of T2D [6]. In addition, *β*-cell deficit of about 20% ∼65% was demonstrated for T2D in several studies [7–9]. Kahn [10] investigated the contribution of insulin resistance and *β*-cell dysfunction to the pathophysiology of T2D. Yoon et al. [11] measured *β*-cell mass in T2D. Although these works intended to study the *β*-cell dysfunction and deficit mechanisms in T2D, they were mainly built upon bulk-cell analysis which can only provide average information about a population of cells.

Since the transcriptomes were firstly measured at single-cell level by Tang et al. in 2009, the technique of single-cell RNA sequencing (scRNA-seq) has experienced an explosive development in the past 10 years [12–15]. Compared with bulk-based approaches, scRNA-seq can provide crucial insights into cellular heterogeneity and bring profound new discoveries in biology [16–21]. For example, Deng et al. reported stochastic expression of monoallelic genes in mammalian cells [22]; Buettner et al. detected hidden subpopulations of cells by analyzing scRNA-seq data [23]. The technique of scRNA-seq has also been applied to transcriptome profiling of human pancreatic cells for both healthy and T2D donors [24–27]. Xin et al. [28] and Segerstolpe et al. [29] showed the expression heterogeneity of human islet cells (e.g. *α*-cells, *β*-cells and *δ*-cells). They also analyzed the alterations of gene expression patterns as well as the enriched signaling pathways in T2D compared with healthy people.

In this study, we aim to unravel the *β*-cell dysfunction and deficit mechanisms in T2D by analyzing the single-cell transcriptomic data of *β*-cells. Three single-cell transcriptomic datasets were adopted because each of the datasets contains more than 100 of *β*-cells (we have found but not used a few other available datasets because they only contain limited numbers of *β*-cells). We named the three datasets as dataset 1, dataset 2 and dataset 3, respectively. All the three datasets consist of *β*-cells obtained from T2D donors and healthy donors. The analysis was carried out from three aspects, i.e. *β*-cell dysfunction, *β*-cell deficit and personalized analysis. Firstly, we focused on the mechanisms of *β*-cell dysfunction in T2D. It is well known that the major role of pancreatic *β*-cells is to produce insulin. Thus, we analyzed the expression levels of INS (i.e. the gene that encodes the preproinsulin precursor of active insulin) in *β*-cells belonging to healthy and T2D donors of each dataset. Different patterns of INS expression were detected in the three datasets. To explore the reasons, we examined the cellular stress in *β*-cells of the three datasets, and applied different machine learning algorithms to discriminate healthy and T2D *β*-cells by using the stress related features. Modeling the vulnerability of T2D *β*-cells to cellular stress, we found that oxidative stress could be a major influential factor on INS expression. Secondly, to study the mechanisms of *β*-cell deficit in T2D, we investigated the expression levels of the genes in the apoptosis pathway, conducted principle component analysis and carried out mutual information calculation. As a result, genes and pathways that are crucial for *β*-cell apoptosis in T2D were detected. In the last part, we performed personalized analysis of INS expression and the expression of death executioner caspases.

Based on the analysis of the three datasets of *β*-cell transcriptomes, we obtained the following main results. Some *β*-cells in T2D donors have comparable INS expression levels with those in healthy donors; *β*-cells in T2D have normal INS expression under low cellular stress, but they have dysfunction under high cellular stress; *β*-cells in healthy people can deal with the cellular stress, maintaining normal INS expression; oxidative stress could be a major influential factor on INS expression; TNFR1-, BAX-, CAPN1- and CAPN2-dependent pathways may be curial for *β*-cell apoptosis in T2D; INS and death executioner caspases are differentially expressed among donors. Note that some of the above results could hardly be obtained from bulk-cell analysis.

## Results

### Pancreatic *β*-cell dysfunction in T2D

#### Single-cell INS expression

**Fig. 1:**
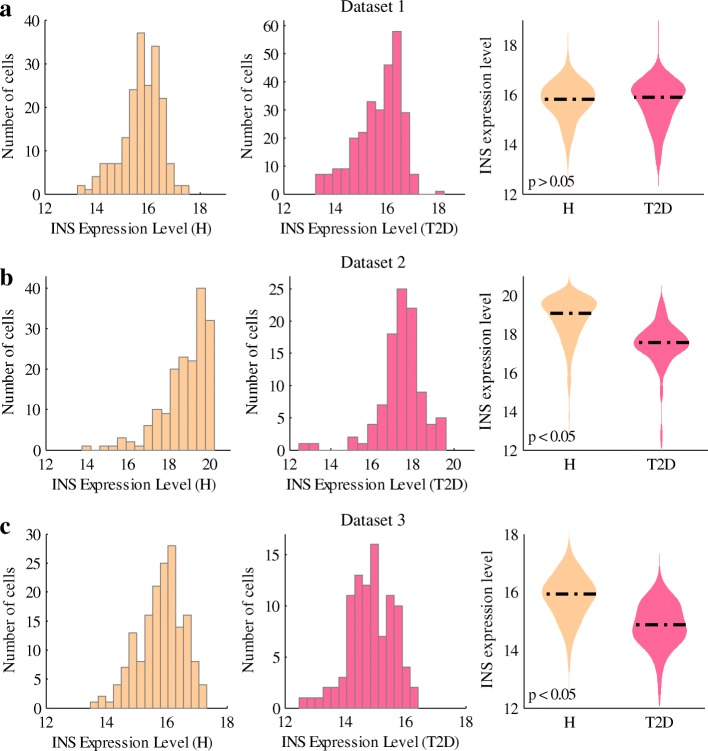
INS expression in the three datasets. Histograms and violin plots are used to show the INS expression levels in *β*-cells of dataset 1 (**a**), dataset 2 (**b**) and dataset 3 (**c**). H stands for healthy. Healthy and T2D samples are colored in light yellow and pink, respectively. A dashed line in the violin plots indicates the median value of each group. *p*-value was calculated by using Student’s *t*-test

In this work, we employed three datasets of *β*-cell transcriptomes, which were obtained from the published works of Xin et al. [28], Segerstolpe et al. [29] and Lawlor et al. [27]. All the three datasets consist of *β*-cells obtained from T2D donors and healthy donors. They were named as dataset 1, dataset 2 and dataset 3, respectively. The numbers of donors and *β*-cells of each dataset are shown in Table 1. Overall, the three datasets comprise gene expression levels of 1,006 *β*-cells from 36 donors. Among the *β*-cells, 472, 270 and 264 belong to the first, second, and third datasets, respectively. Figure 1 presents the INS expression levels of healthy and T2D *β*-cells of the three datasets. As shown in the figure, the single-cell data can reveal cellular heterogeneity in the INS expression, which cannot be obtained from bulk-cell analysis. For both datasets 2 and 3, the median value of INS expression in T2D *β*-cells is lower than that of the healthy *β*-cells, although some T2D *β*-cells have comparable INS expression levels with the healthy ones. In dataset 1, however, the median values of INS expression of healthy and T2D *β*-cells are almost equal. It is well known that one characteristic of T2D is a relative deficiency of insulin. However, this characteristic is not shown in dataset 1. To uncover the regulatory factors of the different INS expression patterns, we investigated the cellular stress in *β*-cells of each dataset, as the increase of cellular stress was detected in T2D by many studies.

**Table 1:** The numbers of donors and *β*-cells of each dataset

Gene expression dataset of *β*-cells		Dataset 1	Dataset 2	Dataset 3
Number of donors	T2D	6	4	3
	Healthy	12	6	5
Number of *β*-cells	T2D	278	99	96
	Healthy	194	171	168

#### Cellular stress

Plenty of evidence indicates that prolonged exposure of *β*-cells to hyperglycemia and high free fatty acids (FFA) causes deleterious effects of endoplasmic reticulum (ER) stress, oxidative stress, and increase of *β*-cell apoptosis [6, 30–34]. ER stress is developed as the continuous demand of insulin, leading to the increased burden of *β*-cell and the accumulation of misfolded proteins in the ER lumen. ER stress is mediated by IRE1, EIF2AK3 and ATF6 [35, 36]. Reactive oxygen species (ROS) are accumulated to cytotoxic level during chronic glucose and fatty acids metabolism [5, 37–39]. Besides, hyperglycemia may disrupt the electron transport chain in mitochondria, which is also a main source of free radicals [40, 41]. The oxidative stress (cumulative ROS) promotes the activation of ASK1, JNK and P38ALPHA. Hyperglycemia and high FFA induce increased *β*-cell apoptosis by several mechanisms, including promoting proapoptotic gene expression, and increasing ER stress, oxidative stress as well as inflammation stress. We use the expression levels of CASP3, CASP6, and CASP7 (i.e. death executioner caspases) to represent the rate of apoptosis.

**Fig. 2:**
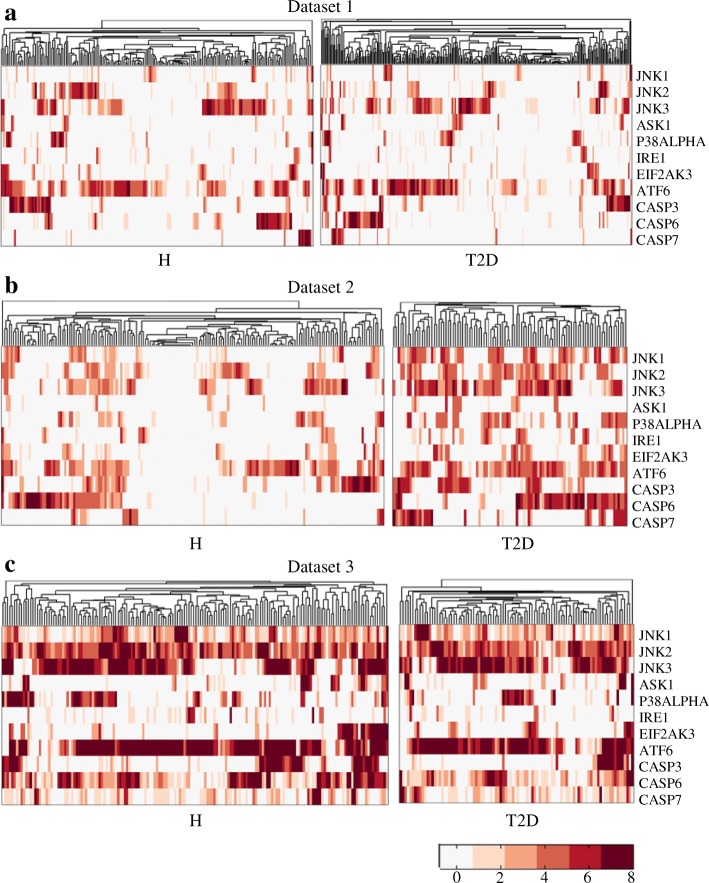
Cellular stress related genes. **a**, **b** and **c** are heatmaps plotting the expression levels of the stress related genes of datasets 1, 2 and 3, respectively. H and T2D represent healthy and T2D *β*-cells. Each row in a heatmap corresponds to a gene and each column represents a cell. Colors in the heatmap denote the log2 expression values

Figure 2 shows the expression of aforementioned stress-related genes, including ER stress, oxidative stress and apoptosis-related genes. As shown in the figure, cellular stress is at a low level for both healthy and T2D *β*-cells in dataset 1 (Fig. 2a), while high stress exists in both groups of cells in dataset 3 (Fig. 2c). In dataset 2, healthy *β*-cells have weak stress whereas T2D *β*-cells experience strong stress (Fig. 2b). By comparing the cellular stresses and the INS expressions of the three datasets (Figs. 1 and 2), the following hypotheses may be proposed: T2D *β*-cells perform similarly to the healthy ones in INS expression under low cellular stress (dataset 1); T2D *β*-cells tend to be dysfunctional under the circumstances of high cellular stress (dataset 2); healthy *β*-cells can partly deal with high cellular stress, maintaining INS expression at normal level (dataset 3, Fig. 1c). To further test these hypotheses, we employed different classifiers to discriminate healthy and T2D *β*-cells.

#### Discrimination of healthy and T2D *β*-cells

In order to test the above analysis results, we employed different classifiers to discriminate healthy and T2D *β*-cells for each dataset, using expression data of genes that are related to ER stress, oxidative stress and apoptosis. In addition, the INS expression related genes were also included as features of the two groups of cells [42, 43]. Overall, 45 genes were selected (Additional file 1: Table S1) [35–41]. Then, we chose genes that were expressed in more than 35% of all the cells in each dataset (40% for dataset 1, these values were derived according to the proportion of healthy *β*-cells in all the cells of each dataset). We conducted this step because a feature (gene) cannot contribute to distinguishing healthy and T2D *β*-cells if it is barely expressed in the cells. Here, expressed genes are those with expression levels no less than 1. Then 17, 25 and 31 genes met the conditions in datasets 1, 2 and 3, respectively. For fair comparison, we also applied the 25 genes selected in dataset 2 to datasets 1 and 3.

**Fig. 3:**
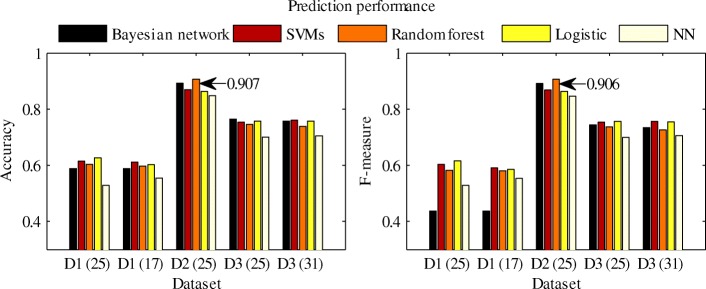
Prediction performance of the five classifiers in discriminating healthy and T2D *β*-cells of each dataset. D1, D2 and D3 represent datasets 1, 2 and 3, respectively. The numbers in the parentheses denote the numbers of genes used as features in the corresponding predictions

Afterwards, five classifiers, i.e. Bayesian network, support vector machine (SVM), random forest, logistic regression and neural network (NN), were used to predict the cellular conditions (i.e. healthy or T2D) of *β*-cells in each dataset. The performance is shown in Fig. 3. Besides prediction accuracy, we also adopted F-measure to evaluate the prediction performance, as the numbers of healthy and T2D cells are not balanced in each dataset. As shown in Fig. 3, all the five classifiers could hardly distinguish healthy and T2D *β*-cells in dataset 1. It implies that healthy and T2D *β*-cells behave similarly in the scenario of low cellular stress. For dataset 2, the prediction accuracy and F-measure are both high, indicating that *β*-cells in T2D have dysfunction under the circumstances of high cellular stress. For dataset 3, the prediction performance is lower than that of dataset 2, probably due to the heavy cellular stress in both of the groups of *β*-cells. However, healthy *β*-cells maintain normal INS expression under high stress (Fig. 1c), suggesting that the cells can partly deal with the cellular stress.

#### Vulnerability of T2D *β*-cells

**Fig. 4:**
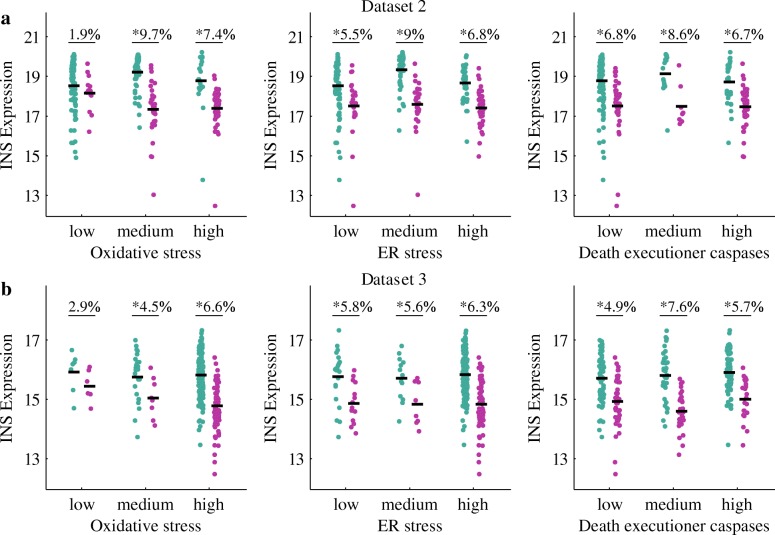
Vulnerability of T2D *β*-cells. **a** and **b** show the INS expression of datasets 2 and 3, under the circumstances of various stresses. Healthy and T2D *β*-cells are colored green and magenta, respectively. The thresholds of 4 and 8 were used to determine different levels of oxidative stress, while the thresholds of 2 and 4 were employed to partition ER stress and death executioner caspases. These thresholds were determined by the expression levels of the genes. The bold dark lines indicate the median values. The extent and significance (* indicates *p*-value <0.05) of the difference between two groups are also provided in the figure. The percentage of difference on the top of each plot was calculated by using the median values of the two groups

In dataset 3, both the healthy and T2D groups of *β*-cells suffer from high cellular stress. However, INS is more highly expressed in healthy *β*-cells than in the T2D ones (Fig. 1c). This is likely due to the fact that pancreatic *β*-cells in T2D are vulnerable to dysfunction as the toxic effects of hyperglycaemia and high FFA (i.e. glucolipotoxicity). In other words, healthy *β*-cells can deal with high cellular stress but T2D *β*-cells cannot, and thereby INS expression is lower in T2D *β*-cells than that in healthy cells under similar cellular stress (Fig. 4). Dataset 1 is not analyzed here, due to the low cellular stress and comparable INS expression between the healthy and T2D groups of *β*-cells. In Fig. 4, oxidative stress, ER stress and death executioner caspases are represented by the expression levels of JNK, ATF6 and CASP6, respectively. We used the three genes as they expressed in more than 35% (the method of deriving the value has been mentioned previously) of all the *β*-cells in each dataset. As shown in Fig. 4a, INS expression in T2D *β*-cells is slightly lower than that in healthy ones when oxidative stress is weak. However, under medium or high oxidative stress, there is a significant difference in INS expression between the two groups. For ER stress and death executioner caspases, INS expression is significantly different between the healthy and T2D cells even when cellular stress is at a low level. Figure 4b shows similar patterns as Fig. 4a, indicating the vulnerability of T2D *β*-cells (as reflected by the reduced INS expression) compared with the healthy *β*-cells under similar cellular stress.

#### Major influential factors for INS expression

**Fig. 5:**
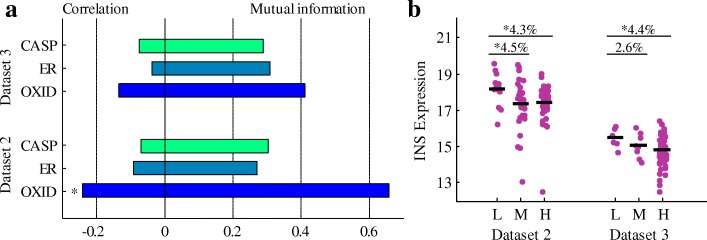
Major influential factors for regulating INS expression. **a** Correlation and mutual information between INS expression and the three factors. OXID, ER and CASP denote oxidative stress, ER stress and death executioner caspases, respectively. **b** INS expression in T2D *β*-cells in the scenarios of low (L), medium (M) and high (H) levels of oxidative stress. The dark horizontal short lines mark the median values. The percentage of difference between two groups is provided in the figure. The symbol of * in both A and B indicates that *p*-value < 0.05

To further determine the major influential factors for INS expression in T2D, we computed the correlation and mutual information between INS expression and the three aforementioned factors (ER stress, oxidative stress and death executioner caspases) for T2D *β*-cells of datasets 2 and 3 (Fig. 5a). As shown in Fig. 5a, the correlation between oxidative stress and INS expression is the strongest, and the mutual information between them is the largest among the three factors. Table 2 lists the detailed values of entropy, joint entropy and mutual information, where INS, ER, OXID and CASP denote INS expression, ER stress, oxidative stress and death executioner caspases, respectively. Entropy and joint entropy measure the average information content of one or a set of variable(s), while mutual information measures the information of one variable obtained through another. Figure 5b shows INS expression in T2D *β*-cells under low, medium and high levels of oxidative stress. For both datasets 2 and 3, there is a significant difference of INS expression between the *β*-cells under low and high levels of oxidative stress. These results reveal that oxidative stress could be a major factor that affects the INS expression.

**Table 2:** Entropy, joint entropy and mutual information

Dataset		2	3
Entropy	INS	1.95	1.65
	OXID	**3.68**	**3.93**
	ER	2.44	3.23
	CASP	2.41	2.85
Joint entropy	INS and OXID	**4.96**	**5.17**
	INS and ER	4.12	4.57
	INS and CASP	4.06	4.21
Mutual information	INS and OXID	**0.66**	**0.41**
	INS and ER	0.27	0.31
	INS and CASP	0.30	0.29

The unit is bit. The bold numbers are the highest values among those compared in the same group (i.e. the same measure and the same dataset)

### *β*-cell apoptosis in T2D

#### Expression of death executioner caspases

**Fig. 6:**
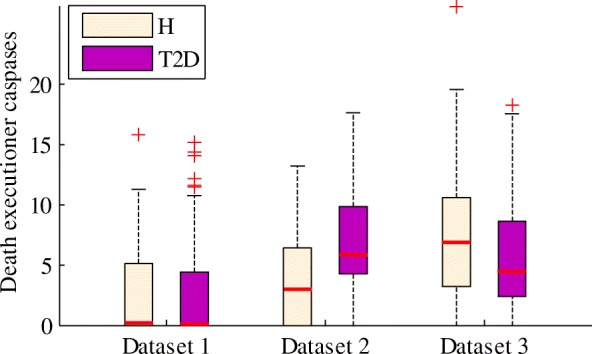
Distribution of the TEDECs (CASP3, CASP6 and CASP7) in each dataset. H represents healthy

In apoptosis, the caspase proteins of CASP3, CASP6 and CASP7 act as death executioner enzymes. Thus, we used the total expression levels of these genes encoding the three caspases to measure the rate of *β*-cell apoptosis. Figure 6 shows the distribution of the total expression of the death executioner caspases (TEDECs) in each dataset. Increased apoptosis in T2D is only observed in dataset 2. In dataset 1, the median values of TEDECs of both groups of cells (i.e. healthy and T2D) approach 0, while the values of the two groups are both high in dataset 3. These patterns are consistent with the cellular stress (i.e., ER stress and oxidative stress) of datasets 1 and 3 (Fig. 2), where low cellular stress is observed in dataset 1 and high stress in dataset 3. Surprisingly, the median value of TEDECs of the healthy *β*-cells is higher than that of the T2D *β*-cells in dataset 3, despite the increased *β*-cell apoptosis and *β*-cell deficit in T2D as reported in the literature. This pattern needs further analysis and explanation in the future.

#### Pathway analysis of apoptosis in T2D

**Fig. 7:**
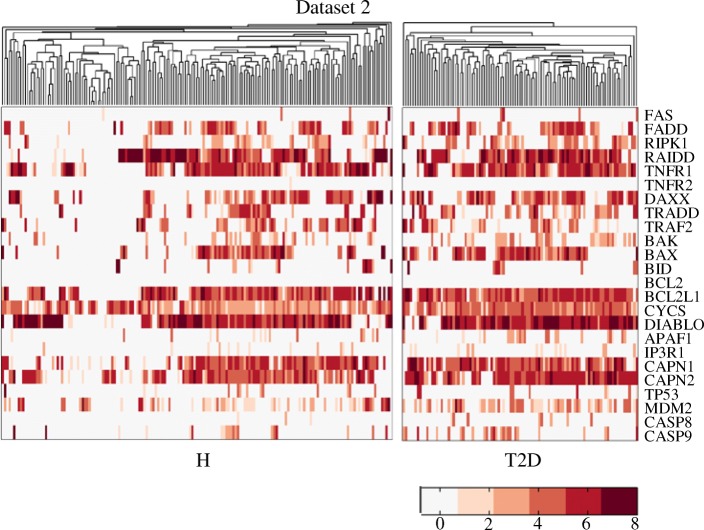
The expression levels of apoptosis-related genes in dataset 2. In the heatmaps, the rows and columns correspond to the genes and cells, respectively. H and T2D represents healthy and T2D *β*-cells. Colors in the heatmaps denote the log2 expression values

To further understand the regulation of abnormal *β*-cell death in T2D, we conducted pathway analysis of apoptosis. Among the three datasets analysed here, the increased level of *β*-cell apoptosis has been only observed in dataset 2 (Fig. 6), thus we focused on dataset 2 to detect the major apoptosis pathway in T2D *β*-cells. According to the apoptosis pathways in KEGG Pathway Database [44] and GeneGO MetaCore [45], a total of 24 apoptosis-related genes were involved in our study. They constitute the death receptor-, mitochondria-, TP53- as well as the ER stressed-mediated apoptosis pathways. The expression levels of these genes are presented in Fig. 7. The genes encoding RIPK1, RAIDD, TNFR1, TRADD, BAX, BCL2L1, CAPN1 and CAPN2 are more highly expressed in T2D *β*-cells than the healthy ones.

**Fig. 8:**
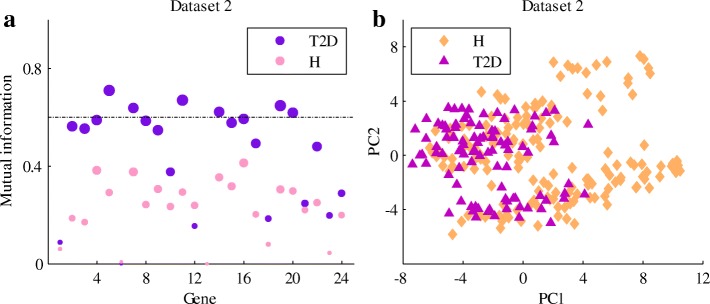
Remarkable genes related to *β*-cell apoptosis in T2D. **a** Mutual information between TEDECs and the genes in the apoptosis pathway. The numbers at x-axis correspond to the order of the genes in Fig. 7. The size of the points represents the amount of mutual information. The dashed line shows the mutual information at 0.6. **b** Projection of *β*-cells (characterized by the genes in the apoptosis pathway) to the first two principal components of the PCA. H represents healthy

We then computed the mutual information between the TEDECs and the proteins in the apoptosis pathway (Fig. 8a, Additional file 2: Table S2). In T2D, much information about the TEDECs can be obtained through the genes encoding TNFR1, DAXX, BAX, BCL2L1, DIABLO, CAPN1 and CAPN2. Besides, we conducted principal component analysis (PCA), and projected the cells (characterized by the 24 apoptosis-related genes) to the first two principal components (Fig. 8b). Although the healthy and T2D groups of cells are not completely separated, they have difference in the new projection. By checking the first two principal components, we found that the values associated with the genes encoding RAIDD, TNFR1, BAX, BCL2L1, DIABLO, CAPN1 and CAPN2 are larger than others (Additional file 3: Figure S1). It implies that these genes contribute significantly to the new projection.

To summarize the above analyses, the death receptor TNFR1-mediated pathway, mitochondrial BAX-related pathway, as well as the CAPN1- and CAPN2-dependent pathway may be crucial in T2D.

We also analyzed the genes related to apoptosis in dataset 3. Different from dataset 2, in dataset 3 TP53 is highly expressed. The results are provided in the supplementary documents (Additional file 2: Table S3, Additional file 3: Figures S2 and S3).

### Personalized analysis of *β*-cells

**Fig. 9:**
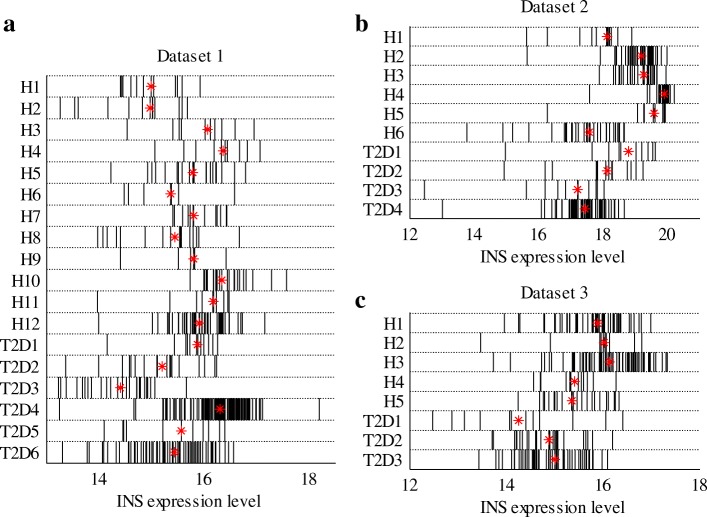
INS expression levels of *β*-cells of each donor. **a**, **b** and **c** show the INS expression in donors of datasets 1, 2 and 3, respectively. H*i* and T2D*i* represent healthy and T2D donors, where *i* stands for the index number of a donor

In addition to comparing the gene expression in healthy and T2D *β*-cells, we also conducted personalized analysis. Figure 9 shows the INS expression of *β*-cells from each donor. Healthy and T2D donors are arranged sequentially in rows. Each vertical bar represents INS expression level of a *β*-cell. The symbol of * indicates the position of the median INS expression level of each donor. As shown in the figure, INS expression is different among donors. In datasets 2 and 3, most of the T2D patients have lower median INS expression levels than the healthy donors.

We also analyzed the TEDECs of each donor (Additional file 3: Figure S4). Similar to the INS expression, the TEDECs are also different among donors. Note that, in dataset 2, the median values of the TEDECs of T2D patients are all larger than those of the healthy donors.

## Discussion

In this work, we conducted single-cell data analysis to decipher pancreatic *β*-cell dysfunction and deficit mechanisms in T2D. Three single-cell transcriptomic datasets were employed in our study. Different from bulk-cell data analysis, single-cell data analysis allows us to capture inter-cell heterogeneity and explore the data deeply to unravel the mechanisms of diseases. It is well known that a major function of pancreatic *β*-cells is to produce secretory insulin. Thus, we firstly examined the INS expression levels in the *β*-cells of each dataset. In datasets 2 and 3, INS expression in the T2D *β*-cells is generally lower than that in the healthy cells, but the expression is similar in healthy and T2D *β*-cells of dataset 1. To explain the observation of INS expression, we checked genes that are related to cellular stress, and found that these genes were lowly expressed in both the healthy and T2D groups of *β*-cells of dataset 1. In dataset 2, T2D *β*-cells had high cellular stress while healthy *β*-cells experienced low stress. Moreover, in dataset 3, the cellular stress in both groups of cells was high.

Considering the INS expression levels and cellular stresses of the three datasets, we obtained the following results. T2D *β*-cells perform normally in INS expression under low cellular stress (dataset 1), but they behave dysfunctionally under high cellular stress (dataset 2); healthy *β*-cells can deal with high cellular stress, maintaining INS expression at normal levels despite the stress (dataset 3). To further validate our analysis results, we employed five classifiers to predict the cellular conditions (i.e. healthy or T2D) of the *β*-cells, using the expression levels of stress- and INS-related genes. We also proposed that *β*-cells in T2D are vulnerable to stress-induced dysfunction. In other words, under similar cellular stresses, T2D *β*-cells have abnormal INS expression while healthy *β*-cells perform normally. This may be caused by the toxic effects of hyperglycaemia and high FFA. Besides, our analysis showed that oxidative stress could be an important influential factor on INS expression. This is consistent with the experimental results in [46], which show that MAFA and PDX1 are inactivated under oxidative stress, resulting in the decrease of insulin secretion of T2D *β*-cells. Meanwhile, the impaired *β*-cell function can be repaired by relieving oxidative stress. For instance, as reported in [47, 48], insulin secretion can be improved in vitro upon treatment with an antioxidant of bis (1-hydroxy-2,2,6,6-tetramethyl-4-piperidinyl) decandioate di-hydrochloride (IAC) in T2D.

T2D is also characterized by a relative deficit of pancreatic *β*-cells [9, 33]. It has long been demonstrated that *β*-cell apoptosis would increase in T2D patients and T2D mouse models [33, 34]. As the apoptosis measurements are not available for the three single-cell datasets, we used the TEDECs (i.e. CASP3, CASP6 and CASP7) to estimate the rate of *β*-cell apoptosis. However, increased apoptosis of T2D *β*-cells is only observed in dataset 2, whereas in dataset 3 the median value of TEDECs of the healthy *β*-cells is higher than that of the T2D *β*-cells. This striking observation needs further clarification as a future work. In addition, we conducted personalized analysis of INS and TEDCEs, and showed that INS and TEDCEs are different among donors, with T2D patients having lower INS expression and higher apoptosis in *β*-cells than healthy donors.

## Conclusions

In this work, to uncover the mechanisms of *β*-cell dysfunction and deficit in T2D, we conducted single-cell transcriptomic data analysis. By analyzing the INS expression levels and cellular stresses of three *β*-cell transcriptomic datasets, we observed that the T2D *β*-cells perform normally in INS expression in the condition of low cellular stress but behave dysfunctionally under high stress. The healthy *β*-cells can deal with high cellular stress and keep INS expression at normal levels. In addition, analyses of correlation and mutual information showed that oxidative stress could be a critical influential factor on INS expression in T2D. This is consistent with some experimental results in the literature. Moreover, we analysed genes related with *β*-cells death regulation and observed increased apoptosis in T2D cells only in dataset 2, when adopting the TEDECs as an estimation of apoptosis rate. The TNFR1-mediated pathway, mitochondrial BAX-related pathway, as well as the CAPN1- and CAPN2-dependent pathway may play important roles in T2D. Finally, personalized analysis showed some diversity of INS expression among donors.

## Materials and methods

### Experimental data of single-cell transcriptomes

The data we analysed here were obtained from three published works of Xin et al. [28], Segerstolpe et al. [29] and Lawlor et al. [27]. The gene expression levels in [28] and [29] were reported in reads per kilobase of transcript per million mapped reads (RPKM), while the records in [27] was quantified as transcripts per million (TPM). Due to the different measurements, we only compared gene expression of cells within the same dataset.

### Classification of cells

In order to predict cellular states (i.e. healthy or T2D *β*-cells), we employed five classifiers: Bayesian network, support vector machine (SVM), random forest, logistic regression and neural network (NN). Bayesian network is a probabilistic graphical model represented by a directed acyclic graph. It contains a set of variables as well as their conditional probability distributions. SVM maps the features into a high-dimensional space and conducts classification using hyperplane(s). Random forest is composed of an ensemble of decision trees, and a voting strategy is employed for the final prediction. In logistic regression, a logistic function is used to compute the probability of the dependent variable and to determine the potential class of a sample. NN is constructed with a group of interconnected neurons, which are organized as the input layer, hidden layer and output layer. Detailed information of these algorithms is provided in [49–54]. We employed the algorithms in Weka 3.8.1 to conduct the classification [55], and leave-one-out cross-validation was used for model validation.

The performance of the algorithms is evaluated using the measurements of accuracy and F-measure. Given the number of instances of true positive (*TP*), true negative (*TN*), false positive (*FP*), and false negative (*FN*), the accuracy is calculated as:

1 $$  Accuracy=\frac{TP+TN}{TP+TN+FP+FN}.  $$

To address the issue that positive and negative samples are not balanced in this study, we also used F-measure which is the harmonic mean of precision and recall. It is calculated as:

2 $$  F - measure=2 \times \frac{presicion\times recall}{precision+recall},  $$

where precision = *TP*/(*TP* + *FP*) and recall = *TP*/(*TP* + *FN*).

### Mutual information

Given two discrete random variables *X* and *Y*, the mutual information provides a measure of the mutual dependence between them [56]. In terms of information, it measures the obtained information about *X* obtained through *Y*, or the uncertainty about *X* reduced through *Y*, and vice versa. The mutual information between *X* and *Y* is defined as follows,

3 $$  I(X;Y)=\sum\limits_{x\in X}\sum\limits_{y\in Y}p(x,y)\log\frac{p(x,y)}{p(x)p(y)},  $$

where *p(x)* and *p(y)* represent the marginal probability distributions, and *p(x,y*) denotes the joint probability distribution. In this work, we calculated the mutual information by using the entropy and joint entropy:

4 $$  I(X;Y)=H(X)+H(Y)-H(X,Y),  $$

where

5 $$  \left\{ \begin{array}{lr} H(X)=-\sum\limits_{x\in X}p(x)\log p(x) & \\ H(Y)=-\sum\limits_{y\in Y}p(y)\log p(y) & \\ H(X,Y)=-\sum\limits_{x\in X}\sum\limits_{y\in Y}p(x,y)\log p(x,y). & \end{array} \right.  $$

*H(X)* and *H(Y)* are the entropies of *X* and *Y*, while *H(X, Y)* stands for the joint entropy of the two variables. The derivation of Eq. (4) from Eq. (3) can be found in [56]. We discretized the gene expression data by taking the floor of the values, as we calculated the entropy in a discrete way. In addition, base 2 was employed for the logarithms to compute entropy, implying that the unit of bit was used for measuring the mutual information.

### Spearman’s rank correlation coefficient

To measure the monotonic relationship between cellular stress and INS expression, we calculated the Spearman’s rank correlation coefficient, following the steps given in [57].

### Principal component analysis (PCA)

PCA was implemented based on an orthogonal linear transformation, which decorrelates samples of possibly correlated variables. After the transformation, the first principle component has the largest variance, the second one holds the second largest variance, and so on. Thus, the fundamental goal of PCA is the change of basis, after which a small number of principal components can be identified to provide a reasonable description of the original data. The derivation and instructions for implementation of PCA are available in [58].

### Comparison of INS expression

We compared the INS expression levels between two groups of cells using Student’s *t*-test. The difference is considered as statistically significant if the *p*-value is less than 0.05.

## Additional files

Additional file 1 Table S1. Genes used in cellular condition (i.e. healthy or T2D) prediction. (XLSX 10 kb)

Additional file 2 Tables S2-S3. Entropy, joint entropy and mutual information between TEDECs and the genes in the apoptosis pathway. (XLSX 13 kb)

Additional file 3 Figures S1-S4. Supplementary figures. (DOCX 742 kb)

## Abbreviations

ER: Endoplasmic reticulum
FFA: Free fatty acids
FN: False negative
FP: False positive
NN: Neural network
PCA: Principal component analysis
ROS: Reactive oxygen species
RPKM: Reads per kilobase of transcript per million mapped reads
scRNA-seq: single-cell RNA sequencing
SVM: Support vector machine
T2D: Type 2 diabetes
TEDECs: Total expression of the death executioner caspases
TN: True negative
TP: True positive
TPM: Transcripts per million
