# Supplementary material for: Single-cell gene expression analysis reveals β-cell dysfunction and deficit mechanisms in type 2 diabetes
Source: BMC Bioinformatics. 2018 Dec 31;19(Suppl 19):515. doi: 10.1186/s12859-018-2519-1 (PMC6311914; doi:10.1186/s12859-018-2519-1)
Supplement: Supplementary file 3 — Figures S1-S4. Supplementary figures. (DOCX 742 kb) [file 12859_2018_2519_MOESM3_ESM.doc]

**Figure S1**

**Figure S1. The first three eigenvectors in PCA analysis of β-cells characterized by the apoptosis-related genes.** The numbers at x-axis correspond to the order of the genes in Figure 7.

**Figure S2**

Dataset 3

FAS

FADD

RIPK1

RAIDD

TNFR1

TNFR2

DAXX

TRADD

TRAF2

BAK

BAX

BID

BCL2

BCL2L1

CYCS

DIABLO

APAF1

IP3R1

CAPN1

CAPN2

TP53

MDM2

CASP8

CASP9

H T2D

0

2

4

6

8

**Figure S2. The expression levels of apoptosis related genes of dataset 3.** H and T2D represents healthy and T2D β-cells. Heatmap was employed, with row and column corresponding to gene and cell, respectively. Colors in the heatmap denote the log_2_ expression values.

**Figure S3**

**Figure S3. Mutual information between the TEDECs and genes in the apoptosis pathway.** H represents healthy. The numbers at x-axis correspond to the order of the genes in Figure 7.

**Figure S4**

**A**

**B C**

**Figure S4. Expression levels of the TEDECs of each donor.** (A), (B) and (C) are violin plots of the TEDECs for dataset 1, dataset 2, and dataset 3. Each violin denotes a donor. H and T2D represents healthy and T2D donors, and they colored deep yellow and light purple, respectively. The bold dark line indicates the median TEDECs value of each donor.
